# Reward-associated distractors can harm cognitive performance

**DOI:** 10.1371/journal.pone.0205091

**Published:** 2018-10-04

**Authors:** Dorottya Rusz, Erik Bijleveld, Michiel A. J. Kompier

**Affiliations:** Behavioural Science Institute, Radboud University, Nijmegen, The Netherlands; University of Wuerzburg, GERMANY

## Abstract

When people carry out cognitive tasks, they sometimes suffer from distractions, that is, drops in performance that occur close in time to task-irrelevant stimuli. In this research, we examine how the pursuit of rewards contributes to distractions. In two experiments, participants performed a math task (in which they could earn monetary rewards vs. not) while they were exposed to task-irrelevant stimuli (that were previously associated with monetary rewards vs. not). In Experiment 1, irrelevant cues that were previously associated with rewards (vs. not) impaired performance. In Experiment 2, this effect was only replicated when these reward-associated distractors appeared relatively early during task performance. While the results were thus somewhat mixed, they generally support the idea that reward associations can augment the negative effect of distractors on performance.

## Introduction

*Distractions*, which we define as performance decrements that occur closely after the onset of a task-irrelevant stimulus, are believed to impair concentration and thwart people’s productivity [1–3]. For instance, interruptions from colleagues harm work productivity [4], using one’s laptop or smartphone during lectures is related to worse academic outcomes [5,6], and using one’s smartphone during driving can lead to fatal consequences [7]. Although the negative consequences are well established, the underlying cognitive/attentional mechanisms of distractions are not yet entirely clear.

In the past, distractions have been mostly seen as originating from a stimulus-driven (i.e., bottom-up) attentional mechanism. That is, stimuli that are physically salient (e.g., because of their abrupt onsets [8] or distinctive colors [9]) are more likely to attract attention–even if these stimuli are irrelevant for the task at hand. This attentional mechanism can explain, for example, why a blinking smartphone screen (with an abrupt onset and distinctive color) attracts attention away from attending to a lecture or driving a car. Recent research, however, shows that physical salience alone may not be able to fully explain distractions. There is rapidly growing evidence that the extent to which task-irrelevant cues grab attention also depends on how much *value* people associate with those task-irrelevant cues [10–14]. In these studies, participants first learned to associate some stimulus features (i.e., color) with the delivery of valuable rewards (i.e., earning money). Later, in a test phase, they performed a visual search task, while the previously reward associated cues reappeared as distractors that needed to be ignored. These studies repeatedly found that participants’ attention was captured by previously-rewarded stimuli, even though these stimuli were completely irrelevant to the task that needed to be done.

While the effect of reward-associated distractors is well established in attentional and visual search tasks (c.f., [15–17]), fewer studies investigated how reward-associated distractors impact other cognitive processes [18,19]. Because real-life tasks (e.g., taking an exam, writing a paper) often involve a large set of cognitive control operations (e.g., maintenance and updating of goal relevant information) beyond visual attention, it is important to investigate whether the impact of reward-related distractors is generalizable across different cognitive operations [20–22]. If this possibility was true, it would suggest that reward-driven distractions have important implications for real-life settings at work, education, and driving, in which optimal performance requires central executive resources [23]. The first aim of this study, therefore, is to expand the existing literature and investigate whether the negative effect of reward-related distractors (i.e., reward-driven distraction) extends to cognitive control operations.

The second aim of this study is to test whether different motivational states influence this reward-driven distraction effect. That is, if the extent to which people get distracted is dependent on how much value they associate with distractors, it should also matter how much value they associate to the *current task*. That is, people are expected to try to optimize performance (i.e., to *exploit*) in a task as long as this task yields more valuable outcomes than its potential alternatives [24,25]. In line with this idea, Müller and colleagues [26] found that monetary incentives can reduce the impact of distractors and help the maintenance of task-relevant information, which leads to better performance. However, when the outcome value of the task decreases, people become less motivated and tend to search for (i.e., to *explore*) alternative behaviors that could provide higher value to them—eventually leading to distraction from the primary task [25]. Based on this line of reasoning, we tested whether distraction by reward-related cues is especially strong in situations when the task does not yield any valuable outcomes–in other words, we predicted that reward-driven distraction is most pronounced when people are not motivated to pursue the current task.

To test these ideas, we developed a new experimental task, building on previous research [10,14]. In short, in the first part of the task, participants learned to associate different colors with monetary (vs. no monetary) rewards. Later, in a second part, they were solving math problems while the previously reward-associated colors reappeared, but this time they had to be ignored. To manipulate participant’s motivational states, some of the math problems were incentivized with monetary rewards. Now, we introduce our experimental task in detail, lay out our specific predictions, and present results from two experiments.

## The experimental paradigm

### Reward learning phase

**Fig 1:**
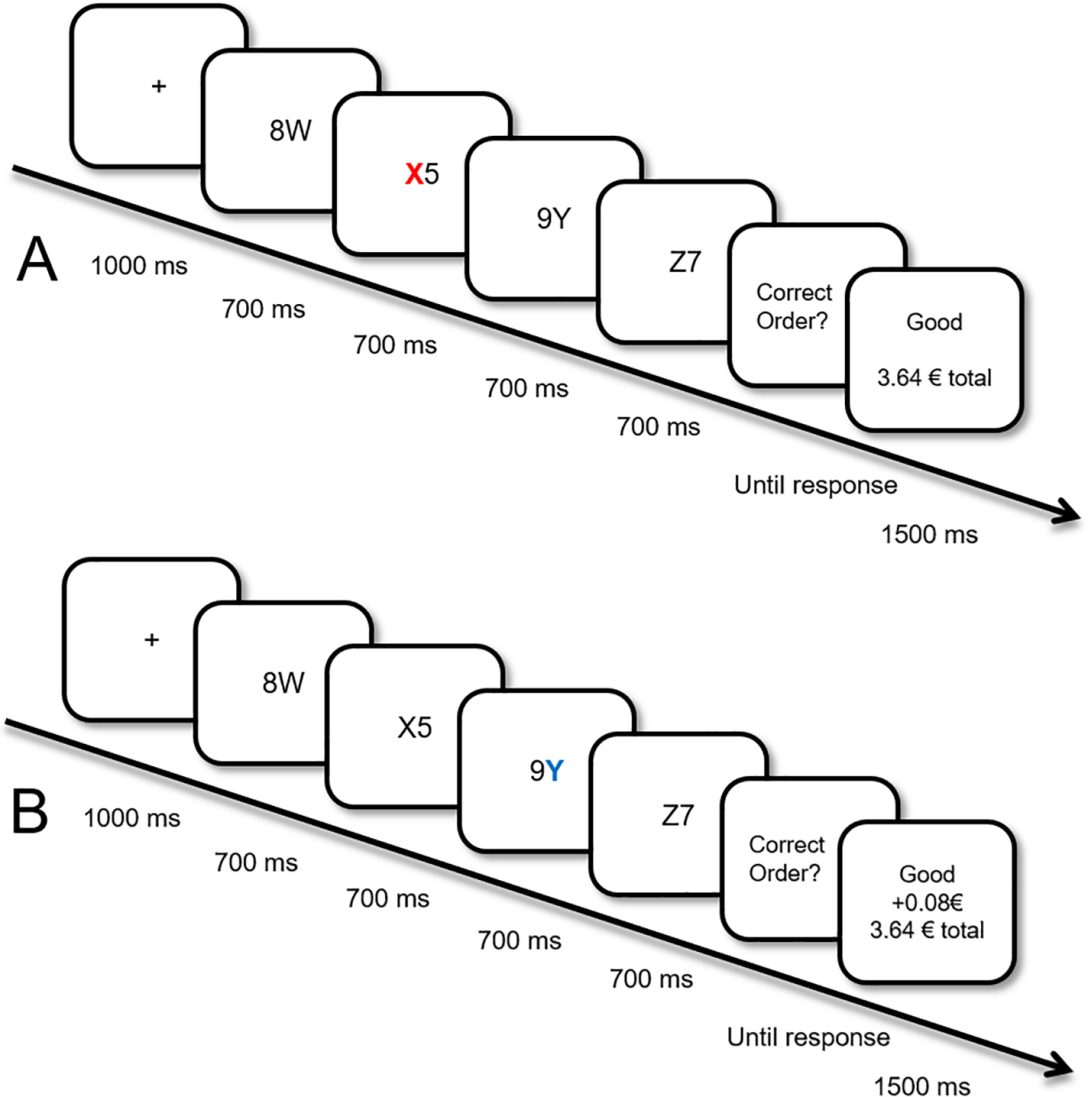
Sequence of events in the training phase. (A) An example of a no reward trial, where the red colored letter “X” predicted no reward. (B) An example of a high reward trial, where the blue colored letter “Y” predicted high reward (8 cents).

In this task, we adopted a well-established reward learning and testing procedure (e.g., [10,11,14]). In the learning phase, each trial consisted of four stimulus-pairs: a letter and a digit presented in close proximity (see Fig 1). Participants’ task was to indicate whether the letters (e.g., W, X, Y, Z) appeared in the correct alphabetical order. One of these letters was always colored in either red or blue. Although participants were told that they could earn money based on correct responses, their reward was also dependent on the colored letter in the sequence. That is, a red letter always predicted earning high rewards (+ 8 eurocents), whereas blue always predicted no rewards (+ 0 eurocents) at the end of the trial (the colors were counterbalanced across participants). We expected that via repeated exposure (150 trials), participants learn to associate rewards to these colors, and these colors, in turn, gain attentional processing priority–a mechanism that has been repeatedly demonstrated in previous research [11,27–30]. In other words, by repeated pairing with reward, these colors would become more salient and therefore would attract attention *more* than other stimuli.

### Reward-driven distraction phase

**Fig 2:**
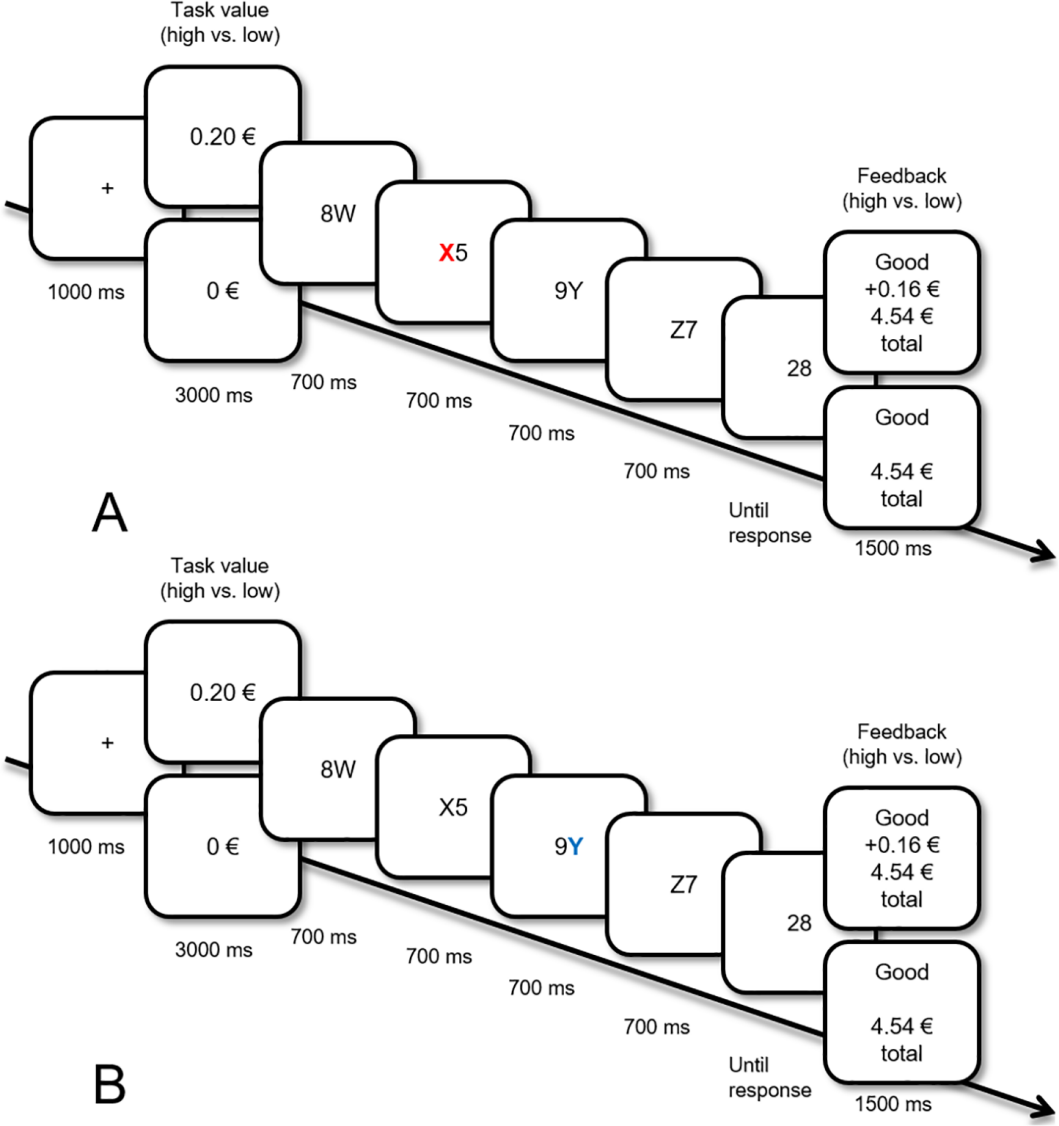
Sequence of events in the test phase. Examples of high or no task value trials with (A) a distractor (e.g., color red “X”) that was previously associated with no reward (B) a distractor (e.g., color blue “Y”) that was previously associated with high reward.

Our main objective was to examine whether these reward-associated cues harm performance in a complex task. For this purpose, we chose a math task that requires a broad set of cognitive functions that people use at work and education [31]. In this phase, participants again saw sequences of four stimulus-pairs: a digit and a letter presented in close proximity (see Fig 2). This time they had to add up the digits and report their sum. Importantly, in the sequence, one of the letters was presented in the previously reward- (vs. no reward) associated color. These colored letters were now task-irrelevant, so they needed to be ignored.

In general, we expected that colored letters that were associated high (vs. no) rewards, would impair performance. Specifically, to get an insight of this performance decrement, we have to zoom in the exact procedure of a trial. First of all, trials were not self-paced, meaning that the digits were presented in a limited time window (700 ms/digit). So, participants had to perform mental additions rather quickly. This was especially demanding during presentation of the second and third stimulus pair, in which participants had to (a) keep mental representations of the targets active (i.e., maintain the sum of the previous digits in working memory), while (b) update this mental representation with new target information (i.e., the next digit in the sequence). Reward-associated distractors appeared during these stimulus pairs. Importantly, as working memory prioritizes processing reward related information [32,33], we expected that previously reward-associated distractors would be prioritized in working memory over the target digits. Consequently, there would be less capacity available to encode target digits, which would weaken the mental representations of these digits and would make it more difficult to update the representation with the subsequent digit, especially given the limited available time. If mental representations would indeed become weaker because of the reward-associated distractors, participants simply would not be able to compute the upcoming mental operation within the allotted time, which would result in an incorrect response. Therefore, we operationalized performance as the percentage of accurate responses on the math task.

We also tested whether reward-driven distraction would be especially strong when people are not motivated to perform the task. In order to test this possibility, we manipulated participant’s motivational states in the test phase by using a monetary reward procedure (e.g., [34–36]). That is, before the trial started, participants were told that they could earn 20 eurocents for a correct response. We expected that promising monetary rewards would induce a high-motivation state, which has shown to boost cognitive resources and effort to perform the task [37–39]. In turn, we expected that this high motivational state would shield mental representations of goal-relevant information from distraction [26,40]. In sum, we expected that high motivational states would suppress reward-driven distraction.

### Hypotheses

In line with decades of research (e.g., [41–44]), we hypothesize that people are more accurate in the math task when they can earn monetary rewards (Hypothesis 1). Second, more importantly, we hypothesize that people are less accurate on the math task when they are exposed to distractors that were previously associated with high (vs. no) rewards (Hypothesis 2). Finally, we hypothesize that people are less accurate when they are exposed to distractors that were previously associated with high (vs. no) rewards, *especially* when their current task does not yield rewarding outcomes (Hypothesis 3).

### Exploring reward-driven distraction

In addition to testing our hypotheses, we also explored two different aspects of our paradigm. First, we explored whether the *timing* of reward-associated distractors mattered. That is, we explored whether disruptions in performance were stronger when the previously reward-associated distractors appeared early (i.e., during the second stimulus pair) vs. late (during the third stimulus pair). Because people actively monitor the time flow of events and update their expectancy about future events [45–47], the timing of distractors may well affect reward-driven distractions.

Second, we explored whether reward-driven distraction influenced performance stability/reliability. That is, on top of traditional performance measures (i.e., response times and accuracy), we computed *performance variability*. Indeed, previous research implies that high motivational states lead to more stable performance (i.e., less fluctuations in performance; e.g., [40,48]). Based on this idea, it is plausible that increased motivation does not just have a general effect on accuracy, but that it reduces the frequency of distractions and thus improves performance stability.

## Experiment 1

### Method

#### Participants and design

This research has been approved by the Ethics Committee of the Social Science Faculty (ECSW2017-0805-50).

Forty-seven students from Radboud University participated in the current study. Students could participate if they (a) slept at least 6 hours during the night before the experiment, (b) were not colorblind, and (c) were native Dutch speakers could participate. After data collection, 3 participants nevertheless reported to have slept less than 6 hours, so they were excluded from the final analysis. Moreover, following similar prior studies (e.g., [27–30]), we excluded 9 participants who performed below 60% accuracy. We did this exclusion to make sure that the final sample consisted only of participants who were capable of performing the task. As such, the final sample consisted of thirty-five students (26 females and 9 males; mean age = 22.3 years, *SD* = 3.7). Participants received compensation in the form of a gift voucher based on their performance (ranging from 7.5–12.5 €). The study used a 2(task value: low vs. high) × 2(distractor value: low vs. high) within-subjects design.

#### Procedure

Participants were seated in a cubicle in front of a computer. First, they signed a consent form and filled out a questionnaire assessing demographics (age, sex), hours of sleep at the previous night, and their need for money on a 1 (*not at all*) to 7 (*very much*) scale (“To what extent are you in need for money at the moment?”). Afterwards, they carried out the task (see below). Finally, they reported on a 1 (*not at all*) to 7 (*very much*) scale how motivated they were and how demanding and difficult they felt the task was (for descriptive statistics, see Table 1). The experiment took 40 minutes to finish. Upon completion of the experiment, they were given the money they earned during the task.

**Table 1:** Descriptive statistics of subjective measures separately for Experiment 1 and Experiment 2.

	Experiment 1		Experiment 2	
Subjective Measures	*M*	*SD*	*M*	*SD*
*Sleep*	7.84	0.76	7.83	0.91
*Task demands*	5.69	1.13	5.35	1.20
*Task difficulty*	4.74	1.36	4.74	1.50
*Fatigue*	4.34	1.08	4.04	1.30
*Motivation*	6.11	0.90	6.11	0.84
*Need for money*	5.0	1.46	4.33	1.58

#### Task

**Stimuli**

The task was designed with E-prime 2.0. Our stimuli were made up of letters and numbers presented in font size 24 in the middle of a monitor screen with a resolution of 1920x1080 pixels.

**Training phase**

Participants first saw a fixation cross, then four sequential displays of a number and a letter presented (e.g., 8W, X5, 9Y, and Z7; see Fig 1). In this phase, letters were the targets and participants were instructed to report whether they were in the correct alphabetical order (e.g., W, X, Y, Z). They responded by pressing “Q” for correct and “P” for incorrect trials. On half of the trials (n = 75), one of the letters had a different color. This colored letter could appear either on the second or the third sub-trial. If this letter was blue (or red, counterbalanced across participants), participants could earn a monetary reward (8 cents). If it was red (or blue, counterbalanced), participants could earn no monetary reward. On low-value trials (e.g., red), responses were followed by visual feedback indicating “Good” or “False”. High-reward trials (e.g., blue) were additionally followed by reward feedback (+ 8 cents) and the total amount that has been earned during the task so far. Participants were not informed about the reward contingency beforehand. There was a 500 ms break in between trials. In total, participants completed 4 practice trials and one block of 150 training trials.

**Test phase**

After the training phase, participants directly started the test phase. First, they received instructions and then immediately started the math task. Participants first saw a fixation cross. Then, they saw the monetary reward that they could earn by responding correctly on that trial (see Fig 2). On half of the trials, participants could earn money (up to 20 cents); on the other half of the trials, they could not (0 cent). Subsequently, participants saw four displays, like in the training phase, each showing a number and a letter (e.g., 8W, X5, 9Y, and Z7). In this part, numbers were the targets and participants were instructed to report whether the sum of the presented numbers (e.g., 8 + 5 + 9 + 7 = 29) was higher or lower than the number presented in the next display (e.g., 28). They responded by pressing “Q” for smaller and “P” for larger sums (29 is bigger than 28, so the correct response would be “P”). On no task value trials, responses were followed by visual feedback indicating “Good” or “False”. High task value trials were additionally followed by reward feedback (e.g., + 16 cents) and the total amount that has been earned during the task so far (e.g., 4.54 €). The amount that could be won on a certain trial decreased with time [36], so fast responses were encouraged. There was a 500 ms break in between trials. Identical to the training phase, on half of the trials, one letter was always red and the other half of the trials one letter was always blue (again, these colored letters could only appear either on the second or the third sub-trial). In this case, the letters served as task-irrelevant stimuli, previously associated with monetary (vs. no monetary) rewards. In total, participants completed 10 practice and one block of 64 test trials (16 trials per condition).

## Results

### Data treatment and performance measures

Responses that were three standard deviations faster or slower than the participant’s mean and responses (based on e.g., [12,49,50]) faster than 300 ms (which were considered guesses) were deleted, which resulted in the exclusion of 5% of trials. For each condition, we computed three performance measures. First, our major performance measure was accuracy, which indicated the percentage of correct responses on the math task. Second, although we did not expect an effect of task value or distractor value on participants’ speed, we explored this variable. So, we computed response times mean (RTM) to explore the average response speed on the math task. Third, we explored performance variability. That is, we computed RT coefficient of variation (RTCV) to assess relative speed variability on the math task–based on suggestions from prior work [40,51]. Neither RTM, nor RTCV were influenced by task and distractor value manipulations (all ps > .05; see descriptive statistics in Table 2, see S1 Appendix for RT analyses, see Table 3 for variability analyses).

**Table 2:** Descriptive statistics of outcome measures separately for Experiment 1 and Experiment 2.

**Experiment 1**		Accuracy (%)		RT (ms)		Variability (%)	
		*M*	*SD*	*M*	*SD*	*M*	*SD*
No Task Value	No Distractor Value	77.7	12.7	996	509	41.94	17.32
	High Distractor Value	74.7	14.9	1008	466	39.67	15.78
High Task Value	No Distractor Value	81.1	11.1	1038	614	38.89	18.75
	High Distractor Value	77.8	14.6	1044	776	36.46	16.01
**Experiment 2**		Accuracy (%)		RT (ms)		Variability (%)	
		*M*	*SD*	*M*	*SD*	*M*	*SD*
No Task Value	No Distractor Value	73.0	14.9	1103	487	41.15	17.36
	High Distractor Value	73.0	15.6	1111	463	41.81	17.89
High Task Value	No Distractor Value	78.5	13.5	1149	517	41.25	19.18
	High Distractor Value	77.5	13.1	1174	564	40.25	16.34

**Table 3:** Results of the experimental effects on accuracy and performance variability both in Experiment 1 and Experiment 2.

**Experiment 1**		Accuracy			Variability		
	dfs	*F*	*p*	η^2^	*F*	*p*	η^2^
1. Task Value	1,34	6.92	.013	.030	2.10	.157	.027
2. Distractor Value	1,34	11.66	.002	.030	2.29	.139	.015
3. Task Value × Distractor value	1,34	.79	.380	.003	.002	.963	< .001
4. Distractor value × Timing	1,34	.98	.329	< .001	.958	.334	< .001
**Experiment 2**							
1. Task Value	1,65	8.65	.005	.001	.139	.711	< .001
2. Distractor Value	1,65	.13	.721	< .001	.016	.899	< .001
3. Task Value × Distractor value	1,65	.22	.644	< .001	.437	.511	< .001
4. Distractor value × Timing	1,65	4.52	.037	.011	.204	.653	< .001

### Confirmatory analyses

**Fig 3:**
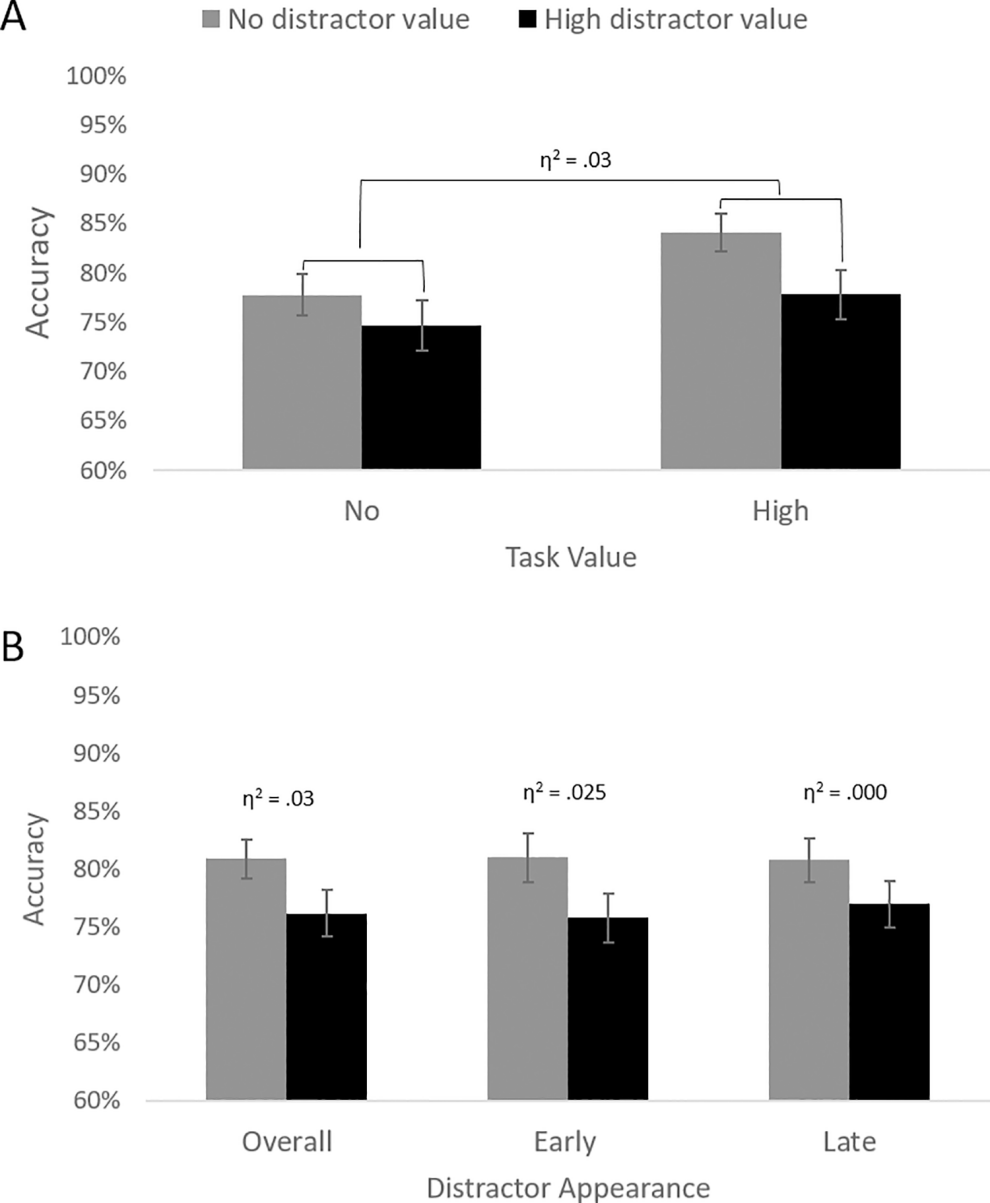
Results of Experiment 1. (A) Accuracy scores for no (gray bars) vs. high (black bars) value distractor trials both in no vs. high task value conditions. (B) Mean accuracy scores by distractor value (high vs. low) on all trials (Overall), on trials where the distractor appeared early, and on trials where the distractor appeared late. Error bars reflect standard errors.

To test Hypotheses 1, 2, and 3, we performed a GLM analysis with task value (high vs. no) and distractor value (high vs. no) as within subject independent variables, and accuracy scores as dependent variable (see Fig 3A). Effect sizes were calculated based on Lakens 2013 [52]. In line with Hypothesis 1, the main effect of task value was significant, *F*(1, 34) = 6.92, *p* = .013, η^2^ = .03, indicating that participants were more accurate when they could earn money (vs. no money; see Table 2 for descriptive statistics). The main effect of distractor value was also significant, *F*(1, 34) = 11.66, *p* = .002, η^2^ = .03, showing that people were less accurate when they were exposed to a high (vs. no) value distractor (in line with Hypothesis 2). The Task value × Distractor value interaction was not significant, *F*(1, 34) = .79, *p* = .380, η^2^ = .003 –thus showing no support for Hypothesis 3.

We further explored whether participants’ (a) need for money (reported in S2 Appendix) and (b) early vs. late distractor appearance affected the results. We corrected for multiple testing by applying Pocock’s boundary [53] for 4 sequential analysis (i.e., same GLM 4 times: people low in need for money, people high in need for money, early trials, and late trials) by lowering the alpha level from 0.05 to 0.0182 –a procedure suggested by [54].

### Exploratory analysis: Distractor timing

We examined whether the timing of (high vs. no reward) distractors in the sequence moderated the effect of distractor value. To test this, we performed the same GLM analyses as above, but now also including Distractor timing (early vs. late) as an additional within-subjects predictor. We specifically examined the Distractor timing × Distractor value interaction, *F*(1, 34) = .98, *p* = .329, η^2^ < .001, which was not significant (see Fig 3B). So, we found no clear evidence for the idea that high (vs. no) value distractors affected performance differently based on whether it appeared early or late.

For consistency with Experiment 2 (see below), we further explored the data and ran our original GLM with a particular interest for the main effect of distractor value, separately for trials in which the distractor appeared early (i.e., in the second stimulus screen) vs. late (i.e., in the third stimulus screen). On *early* distractor trials, participants were less accurate on high (*M* = 76%, *SD* = 16%) vs. no reward distractor (*M* = 81%, *SD* = 16%) conditions, *F*(1, 34) = 6.21, *p* = .018, η^2^ = .025. On *late* distractor trials, the main effect of distractor value was not significant, *F*(1, 34) = 3.30, *p* = .078, η^2^ < .001.

## Discussion

The results of Experiment 1 provide initial evidence for a motivational perspective on distraction. In line with Hypothesis 1, we found that people were more accurate when they could earn money on the task. This is consistent with the idea that motivation (e.g., monetary incentives) boost cognitive control processes that lead to better performance on cognitive tasks [38].

In line with Hypothesis 2, we found that people were less accurate when they were exposed to irrelevant cues that were previously associated with high (vs. no) reward. This finding supports the idea that (a) distraction may be reward-driven [15,17] (b) that reward-associated distractors interfere with the active, ongoing maintenance of task relevant information and thus impair cognitive performance. We wanted to better understand *when* these reward-associated distractors harm task performance the most. So, we further explored whether the timing of distractors moderated this reward-driven distraction effect, but found no evidence for the possibility.

Finally, contrary to Hypothesis 3, we found that participants were no more likely to be distracted (by high-reward distractors) when they were in a high (vs. low) motivational state. In order to investigate whether the results were replicable, we conducted another experiment on an independent sample. Before the start of data collection, we preregistered Experiment 2, a direct replication of Experiment 1 at the Open Science Framework (https://osf.io/y74kx/). Specifically, we pre-registered our hypotheses, the planned sample size, and the analysis plan. As it is important to distinguish between analyses that were planned before vs. not [55], we present analyses that were preregistered as *confirmatory* and analyses that were not preregistered as *exploratory*.

## Experiment 2

### Method

The design, procedure, and task were identical to Experiment 1. The preregistration, experimental materials, and the data can be found in OSF (https://osf.io/y74kx/). We conducted a power analysis with Glimmpse [56], which suggested that a sample size of N = 54 should be sufficient to detect all effects of interest with power = .90. Because of our rather strict exclusion criteria (see below), we wanted to be on the safe side. Therefore, we recruited seventy-three students from Radboud University. The same a priori exclusion criteria were applied as in Experiment 1. One participant reported to sleep less than 6 hours during the night before the experiment and five participants performed below 60% accuracy, so they were excluded from the final analysis. The final sample consisted of sixty-six students (50 females and 16 males; mean age = 23.4, *SD* = 5.4). For descriptive statistics of subjective measures, see Table 1. Participants received monetary compensation in the form of a gift voucher based on performance (ranging from 7.5–12.5 €).

### Results

**Fig 4:**
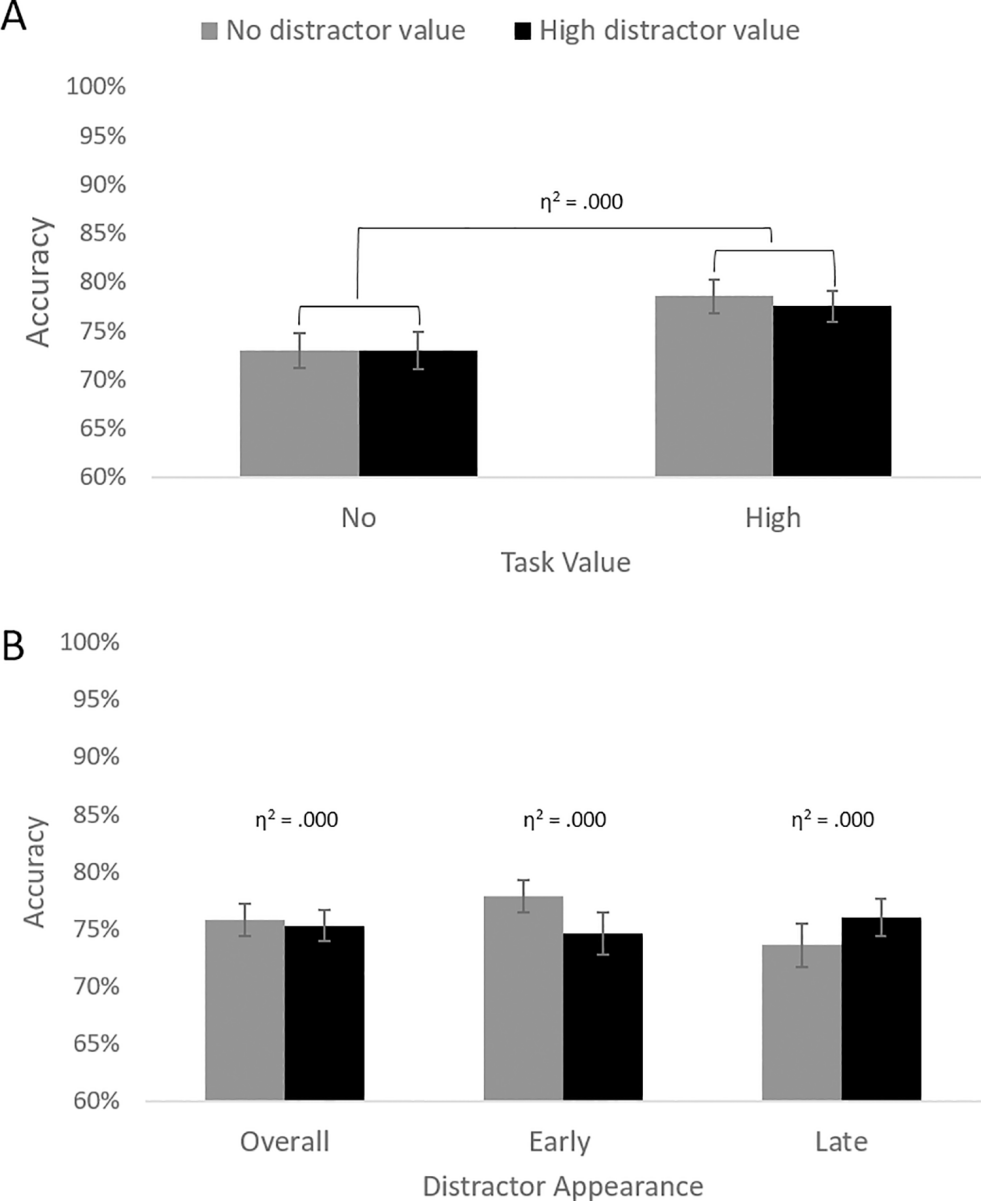
Results of Experiment 2. (A) Accuracy scores for no (gray bars) vs. high (black bars) value distractor trials both in no vs. high task value conditions. (B) Mean accuracy scores by distractor value (high vs. no) on all trials (Overall), on trials where the distractor appeared early, and on trials where the distractor appeared late. Error bars reflect standard errors.

Responses that were three standard deviations faster or slower than the participant’s mean and responses faster than 300 ms (which were considered guesses) were deleted, which resulted in the exclusion of 2% of trials. As pre-registered, to test Hypotheses 1, 2, and 3, we performed a GLM analysis with task value (high vs. no) and distractor value (high vs. no) as within-subject independent variables, and accuracy scores (percentage of correct responses) as the dependent variable (see Fig 4A).

#### Confirmatory analyses

In line with Hypothesis 1, replicating results of Experiment 1, the main effect of task value was significant, *F*(1, 65) = 8.65, *p* = .005, η^2^ = .001, indicating that participants were more accurate when they could earn money (vs. no money; see Table 2 for descriptive statistics). Unlike in Experiment 1, the main effect of distractor value was not significant, *F*(1, 65) = .13, *p* = .721, η^2^ < .001,–showing no support for Hypothesis 2. As in Experiment 1, the interaction effect was also not significant, *F*(1, 65) = .22, *p* = .644, η^2^ < .001 (i.e., no support for Hypothesis 3).

Exploratory analyses on participants’ response times and need for money are in S1 Appendix and S2 Appendix. Like in Experiment 1, we corrected for multiple testing by applying Pocock’s boundary [53] for 4 sequential analysis (i.e., same GLM 4 times: people low in need for money, people high in need for money, early trials, and late trials) by lowering the alpha level from 0.05 to 0.0182.

#### Exploratory analysis: Distractor timing

We ran our original GLM, but now adding distractor timing as a factor. The Distractor timing × Distractor value interaction was significant, *F*(1, 65) = 4.52, *p* = .037, η^2^ = .011, suggesting that the timing of the distractor moderated the effect of distractor value. Follow up analyses revealed that, the main effect of distractor value was neither significant on early-distractor trials, *F*(1, 65) = 3.47, *p* = .067, η^2^ < .001, nor on late-distractor trials, *F*(1, 65) = 1.34, *p* = .251, η^2^ < .001.

**Fig 5:**
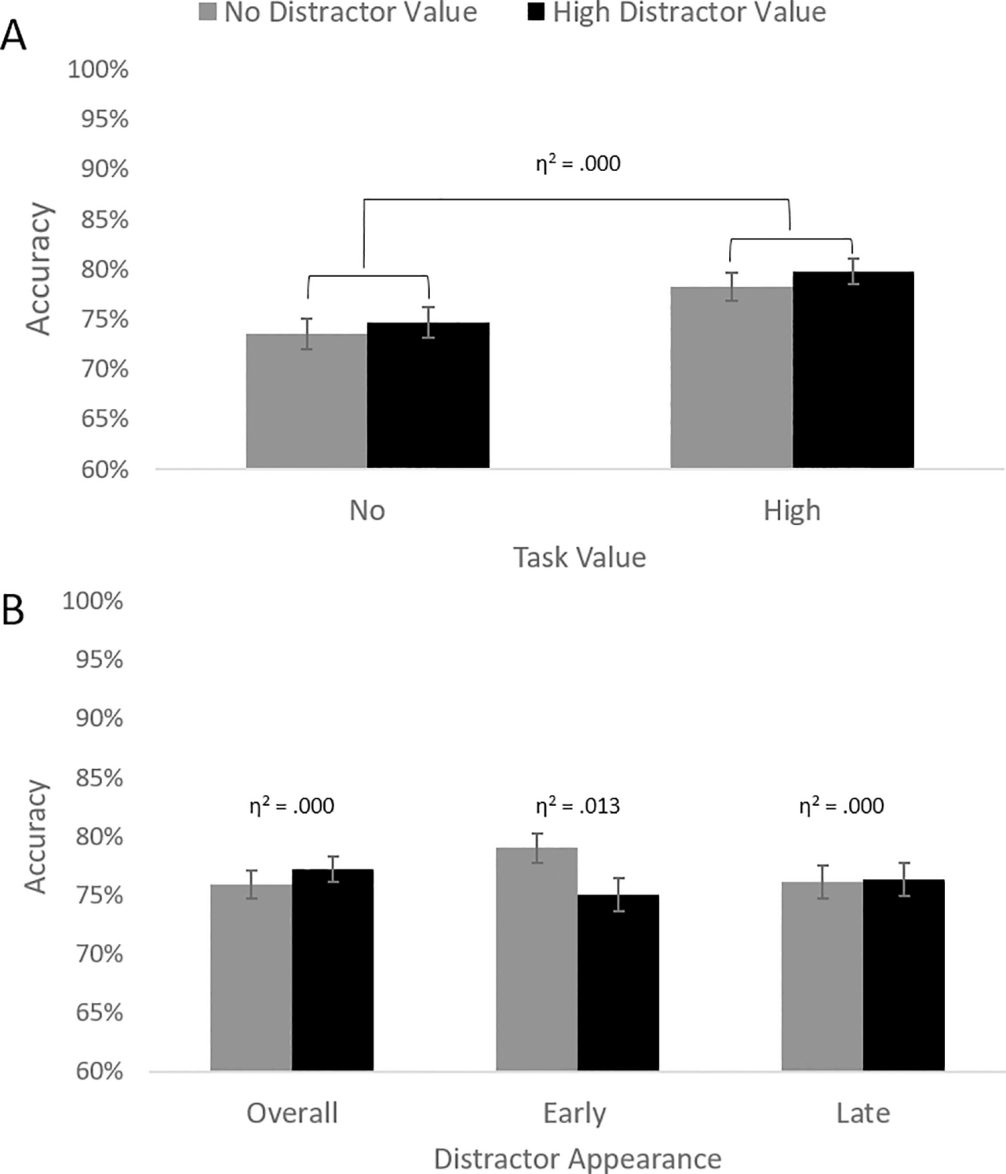
Pooled data from Experiment 1 and Experiment 2. (A) Accuracy scores for no (gray bars) vs. high (black bars) value distractor trials both in no vs. high task value conditions. (B) Mean accuracy scores by distractor value (high vs. low) on all trials (Overall), on trials where the distractor appeared early, and on trials where the distractor appeared late. Error bars reflect standard errors.

In short, in Experiment 2, while the Timing × Distractor value interaction was significant, we found no main effect of distractor value separately in early and late timing trials. On the contrary, in Experiment 1, the Timing × Distractor value interaction was not significant, but the early impact of distractors seemed stronger than late. To provide the most reliable effect size estimates we can provide at this point, we re-ran the analysis on the pooled data from both experiments, a procedure suggested by Lakens [57]. We explored the Distractor timing × Distractor value interaction, which was significant, *F*(1, 100) = 4.21, *p* = .043, η^2^ = .006. Inspection of Fig 5B suggests that the effect of distractor value was the largest for early distractors (η^2^ = .013), compared to late distractors late (η^2^ < .001). So, considering both studies together, we found some support for the possibility that early high-reward distractors had a stronger impact than late high-value distractors. We note that this finding should be interpreted with caution, as these analyses were not pre-registered and because the effect size was small.

### Discussion

The purpose of Experiment 2 was to replicate the results of Experiment 1. As in Experiment 1, people were more accurate when they could earn money in the task (Hypothesis 1). Unexpectedly, however, we did not replicate the negative effect of reward-associated distractors on performance (Hypothesis 2), i.e., people were not less accurate when they were exposed to distractors that carried high (vs. no) value. As this null effect was somewhat surprising given the strength of the effect in Experiment 1 (η^2^ = .03), and given the fact that the task was identical, we again explored whether early vs. late distractors have had different effects on accuracy, but now on the pooled sample (N = 101). We found that the impact of reward-associated distractors was more pronounced when it appeared early vs. late in the math sequence. Although this analysis was not pre-registered (and should thus be interpreted with caution), this finding suggests that the effect of distractor value may be more pronounced in the early stages of task performance (for interpretations, see General Discussion). In Experiment 2, like in Experiment 1, we did not find that people’s current motivational state affected the impact of high-value distractors; thus, findings do not provide support for Hypothesis 3.

## General discussion

In this research, we had two major aims: (a) to test whether reward-associated distractors harm cognitive control processes and (b) to test whether this reward-driven distraction effect can be eliminated by high motivational states. Two identical experiments yielded strong evidence for the positive effect of monetary incentives on cognitive performance (Hypothesis 1), some evidence that reward-associated distractors disrupt cognitive control processes (Hypothesis 2), and no evidence that high motivational states (i.e., promising monetary rewards) reduce reward-driven distraction (Hypothesis 3). We will discuss each of these findings in detail below.

In line with Hypothesis 1, both experiments showed that people were more accurate in solving mental additions when they could earn money. This finding is in line with the well-established idea that monetary incentives boost cognitive processes (c.f., [37,38,41]), particularly the active maintenance of information in working memory [58,59].

On the contrary, evidence for reward-driven distraction (Hypothesis 2) was somewhat mixed across studies. In Experiment 1, we found direct support for this hypothesis: people were more distracted by high (vs. no) reward-associated distractors–independently of whether distractors appeared early vs. late during task performance. However, in Experiment 2, support for Hypothesis 2 was less clear: the timing of reward-associated distractors moderated the distraction effect. When we explored this idea further in a combined analysis of both studies in order to get the most reliable effect size [52], we found that early distractors were indeed more harmful than late distractors (η^2^ = .013 vs. η^2^ = .000; see below for interpretations). Yet, as the latter finding was done using a non-pre-registered analysis, it should be interpreted with some caution. In sum, results from two studies provide preliminary support for the idea that (a) irrelevant, but rewarding cues may disrupt cognitive control processes and (b) that this effect may be stronger when distractors appear early in a sequence of cognitive control operations. This finding extends prior research and shows that reward-associated distractors do not only slow down visual search [11,13], but they likely interrupt more complex cognitive control operations (i.e., maintenance and updating task-relevant information). These conclusions are consistent with growing literature that distractions may stem from a reward-driven mechanism [15,39].

Reward-driven distraction, in this study, seemed to be influenced by the timing of distractor in the math sequence. More specifically, we expected that high (vs. no) reward-associated distractors will gain priority in working memory over goal-relevant information [32,33], which will weaken the mental representations of targets, which will result in incorrect responses. Unexpectedly, this effect seemed strongest when high reward associated distractors appeared early (vs. late) in the math sequence. This finding could be explained by *conditional probability monitoring* [45–47,60,61]. Specifically, *conditional probability monitoring* is the phenomenon that people continuously monitor the flow of events, and update their expectancy about upcoming events; this expectancy, in turn, affects how they deal with future, unexpected events. Applying this to our paradigm, it seems likely that participants learned that each trial contained a distractor. Also, participants may have learned that when the distractor did *not* appear early (i.e., in the 2^nd^ stimulus pair), it definitely had to appear late (i.e., in the 3^rd^ stimulus pair). As a result, when the distractor appeared late, participants had the opportunity to prepare for it helping them to shield goal-relevant information from the reward-associated distractor. Conversely, such preparation could not happen when the distractor appeared early. As pooled data from both experiments were in line with this explanation, it would be interesting to test this idea in a confirmatory manner. Such confirmatory work would shed more light on the circumstances under which reward-associated distractors disrupt cognitive control processes. Conditional probability monitoring may well be part of the explanation.

Although participants performed better when they could earn monetary rewards on the task, both experiments showed no evidence for Hypothesis 3 –i.e., the prediction that reward-driven distraction would be the strongest when participants are not motivated to perform the task. This is somewhat surprising, given that several contemporary models of motivation and task performance suggest that people’s performance is determined by a computation between the value of the outcomes of the present task and the value of alternatives [62–64]. Also, we expected that higher motivational states would help protect mental representations of goal-relevant information [26,40] from reward-associated distracting information. By contrast to these ideas, our findings suggest that task-irrelevant stimuli (that are associated with rewards) may impact performance independently of whether people are currently motivated to perform well. Future research is necessary to better understand whether—and, if so, under what conditions—rewards for current task can shield people from distractions. Perhaps it may have been confusing for participants that both the value of distractors and the value of the task was manipulated with monetary rewards. To circumvent this issue, future studies could apply simple “try harder” instructions, which has been shown to be efficient in inducing stable performance, which shields against the impact of distractors [40].

Adopting a reward-driven perspective on distraction [14] has implications for practice. First, optimal performance at work and school is known to rely on central executive resources [23], which we found to be disrupted by task-irrelevant stimuli associated with rewards. Smartphones may be an instance of such stimuli: at least, smartphones are pervasive sources of distraction [65] that indeed interfere with work [66] and study [5]. Assuming that smartphones have rewarding properties [67], this way of thinking about smartphones—i.e., as reward-related distractors—may support new models of smartphone-related behavior (e.g., smartphone addiction could be conceptualized, and treated, as a condition similar to Gambling Disorder)

### Strengths and limitations

Throughout this project, we aimed to work in an open and transparent way, in line with recent discussions in psychology [55,68,69]. Specifically, we preregistered the second experiment and tried to directly replicate our results, aiming to actively avoid drawing false conclusions that would eventually distort the literature on the topic [70].

However, in this replication attempt (Experiment 2) we found evidence for reward-driven distraction only when the distractor appeared early in the math sequence. Thus, we have to be careful with drawing strong conclusions. Although, it is plausible that out of multiple studies testing the same hypotheses, some tests turn out to be non-significant [57], the inconsistency between studies is surprising as the effect of rewarding distractors on attentional capture has been well established by prior work [15]. We should mention, however, that our experiments are different from most prior experiments [11,14] in this field in two major ways. Below, we address these two aspects and provide methodological suggestions.

First, unlike previous experiments, we manipulated participants’ current motivational state in the test phase (to test Hypotheses 1 and 3). Specifically, in half of the trials, people could earn money for performing well. Possibly, these performance incentives affected people’s motivational state throughout the task, on all trials, in a sustained way (e.g., [51]). Very speculatively, such sustained changes in motivational state may have reduced the potency of high-value distractors. In future research, it may be promising to solely examine the effect of rewarding distractors, independently of current motivational states.

Second, an important difference between the present research and previous experiments [14] in this field concerns the spatial position of target and distractor. Although previous studies used a search task, in which stimuli are typically located far apart, we used a task in which target and distractor were close next to each other, in the center of the screen. Importantly, this central part of the visual field is processed most efficiently [71]. This enhanced processing efficiency can potentially explain why our task may be less sensitive to the effects of high-value distractors. To further investigate this possibility, future studies may use a task in which the location of target and distractor is farther from each other, more like in visual search paradigms.

### Concluding remarks

The present research provides some first steps in investigating when and how rewarding irrelevant cues disrupt executive control processes. We found that people sometimes perform worse on a math task when they are exposed to a stimulus that was previously-rewarded (vs. not rewarded) and when this stimulus appears early during task performance. This effect was not moderated by people’s current motivational states. Our studies join a growing body of literature [11,14,19,59] that suggests that it may be fruitful to think of distractions from a reward-driven perspective.

## Supporting information

S1 Appendix Exploratory analysis on participants’ response times both in Experiment 1 and Experiment 2. (DOCX)

S2 Appendix Exploratory analysis on participants’ need for money both in Experiment 1 and Experiment 2. (DOCX)
