# Supplementary material for: Reward-associated distractors can harm cognitive performance
Source: PLoS One. 2018 Oct 4;13(10):e0205091. doi: 10.1371/journal.pone.0205091 (PMC6171909; doi:10.1371/journal.pone.0205091)
Supplement: S1 Appendix — (DOCX) [file pone.0205091.s001.docx]

**S1 Appendix. Exploratory analysis on participants’ response times both in Experiment 1 and Experiment 2.**

**Experiment 1**

For exploratory purposes we performed a GLM analysis with task value (high vs. no) and distractor value (high vs. no) as within subject independent variables, and response times as dependent variable (see Table 2 for descriptive statistics). The main effect of task value was not significant, *F*(1, 34) = .63, *p* = .433, η_p_^2^ = .02. The main effect of distractor value was also not significant, *F*(1, 34) = .15, *p* = .705, η_p_^2^ = .00. The task value × distractor value interaction was not significant, *F*(1, 34) = 00, *p* = .947, η_p_^2^ = .00.

**Experiment 2**

For exploratory purposes we performed a GLM analysis with task value (high vs. no) and distractor value (high vs. no) as within subject independent variables, and response times as dependent variable. The main effect of task value was marginally significant, *F*(1, 65) = 3.88, *p* = .054, η_p_^2^ = .06. The main effect of distractor value was not significant, *F*(1, 65) = .59, *p* = .445, η_p_^2^ = .01. The task value × distractor value interaction was not significant, *F*(1, 65) = .21, *p* = .649, η_p_^2^ = .00.
