# Supplementary material for: Reward-associated distractors can harm cognitive performance
Source: PLoS One. 2018 Oct 4;13(10):e0205091. doi: 10.1371/journal.pone.0205091 (PMC6171909; doi:10.1371/journal.pone.0205091)
Supplement: S2 Appendix — (DOCX) [file pone.0205091.s002.docx]

**S2 Appendix. Exploratory analysis on participants’ need for money both in Experiment 1 and Experiment 2.**

**Experiment 1**

To investigate how value-driven distraction is different for people who are low vs. high in need for money we performed the same GLM as above, now also adding need for money (standardized) as a continuous independent variable. This yielded a non-significant Task value × Distractor value × Need for money three-way interaction, *F*(1, 33) = 4.09, *p* = .051, η_p_^2^ = .11.

To explore the nature of this (non-significant, but moderate-to-large effect size, η_p_^2^ = .11) three-way interaction we conducted the same GLM separately for a typical person low in need for money (i.e., an individual 1 *SD* below the mean) and for a typical person high in need for money (i.e., an individual 1 *SD* above the mean; a procedure suggested by Cohen, Cohen, West, and Aiken (2013)).

For people who were low in need for money the analysis revealed a main effect of task value, *F*(1,33) = 6.20, *p* = .018, η_p_^2^ = .16, indicating that people were more accurate when they could earn money (vs. no money) on the trial. There was a significant main effect of distractor value, *F*(1,33) = 14.98, *p* < .001, η_p_^2^ = .31, indicating that participants were less accurate when they were exposed to a high value (vs. low value) distractor. The interaction was not significant, *F*(1,33) = .62, *p* = .436, η_p_^2^ = .02. Yet, the effect of distractor value seemed especially pronounced in the low task value condition, *F*(1,33) = 13.17, *p* = .001, η_p_^2^ = .29, rather than in the high-task value condition, *F*(1, 33) = 2.31, *p* = .138, η_p_^2^ = .07.

For people who were high in need for money, the analysis revealed neither a main effect of task value, *F*(1, 33) = 1.43, *p* = .241, η_p_^2^ = .04, nor a main effect of distractor value, *F*(1, 33) = 1.24, *p* = .273, η_p_^2^ = .04. The Task value × Distractor value interaction was also not significant, *F*(1, 33) = 4.37, *p* = .044, η_p_^2^ = .12. The effect of distractor value seemed more pronounced in the high task value condition, *F*(1, 33) = 4.13, *p* = .050, η_p_^2^ = .11, rather than in the low-task value condition, *F*(1, 33) = 1.39, *p* = .246, η_p_^2^ = .04.

**Experiment 2**

As we proposed in the preregistration, we investigated whether value-driven distraction is different for people who are low vs. high in need for money. Thus, we performed the same GLM as above, but now also added need for money (standardized) as a continuous independent variable. This yielded a non-significant Task Value × Distractor Value × Need for Money three way interaction *F*(1, 64) = 2.56, *p* = .115, η_p_^2^ = .04. To explore the nature of this three-way interaction we conducted the same GLM separately for a typical person low in need for money (i.e., an individual 1 *SD* below the mean) and for a typical person high in need for money (i.e., an individual 1 *SD* above the mean).

For people who were low in need for money, there was no main effect of task value, *F*(1,64) = .81, *p* = .371, η_p_^2^ = .01. There was also no main effect of distractor value, *F*(1, 64) = .03, *p* = .854, η_p_^2^ = .00. The interaction effect was also not significant, *F*(1, 64) = .65, *p* = .424, η_p_^2^ = .01. The effect of distractor value was not significant in the low task value condition, *F*(1, 64) = .45, *p* = .505, η_p_^2^ = .01, and also not significant in the high task value condition, *F*(1, 64) = .12, *p* = .726, η_p_^2^ = .00.

For people who were high in need for money, there was a significant main effect of task value, *F*(1, 64) = 10.90, *p* = .002, η_p_^2^ = .15, indicating that people were more accurate when they could earn money (vs. no money). There was no main effect of distractor value, *F*(1,64) = .10, *p* = .753, η_p_^2^ = .00. We found a non-significant task value × distractor value interaction, *F*(1,64) = 2.13, *p* = .148, η_p_^2^ = .03. Further analysis revealed that the effect of distractor value was neither significant in the high task value condition, *F*(1, 64) = .49, *p* = .488, η_p_^2^ = .01, nor in the low task value condition, *F*(1, 64) = 1.29, *p* = .261, η_p_^2^ = .02.
